# Supplementary material for: Exploring the Therapeutic Potential of Ampelopsis grossedentata Leaf Extract as an Anti-Inflammatory and Antioxidant Agent in Human Immune Cells
Source: Int J Mol Sci. 2023 Dec 28;25(1):416. doi: 10.3390/ijms25010416 (PMC10779184; doi:10.3390/ijms25010416)
Supplement: Supplementary file 1 [file ijms-25-00416-s001.zip › ijms-2783753-supplementary.pdf]

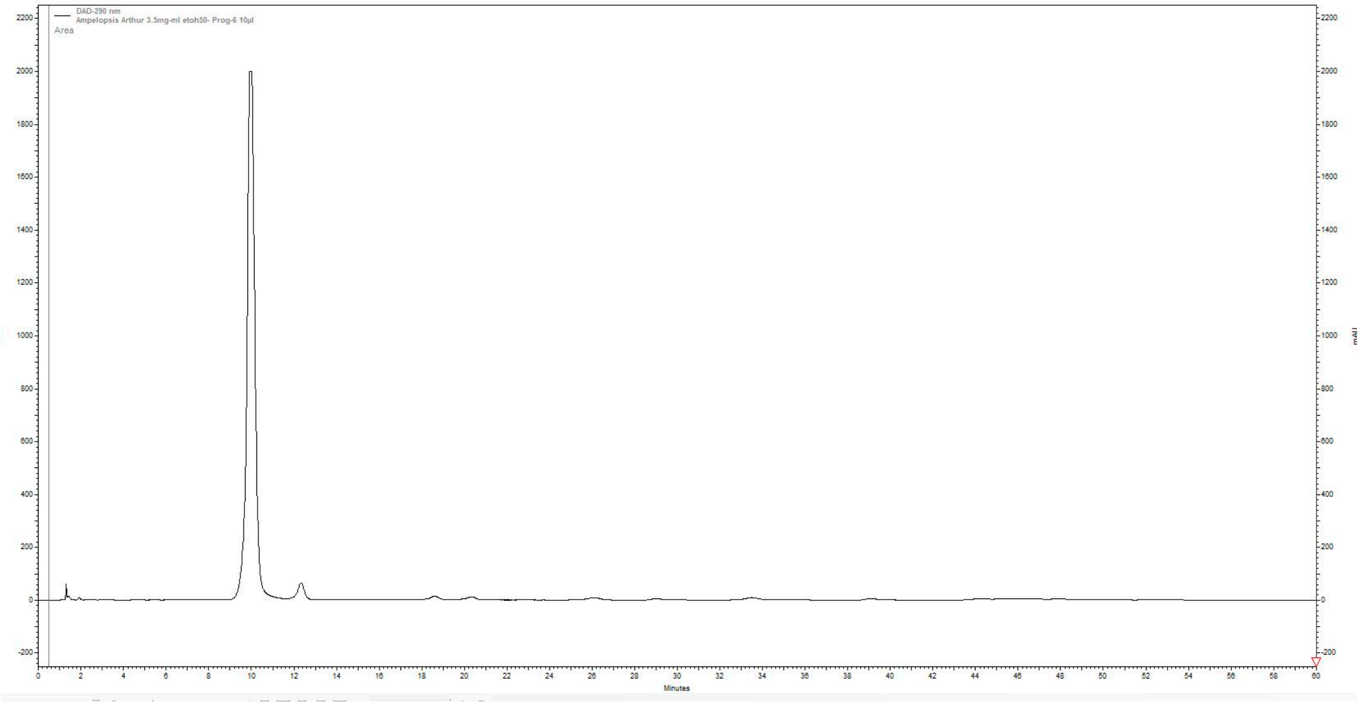

Figure S1 : Quantification of Dihydromyricetin (DHM) Content using HPLC-UV.

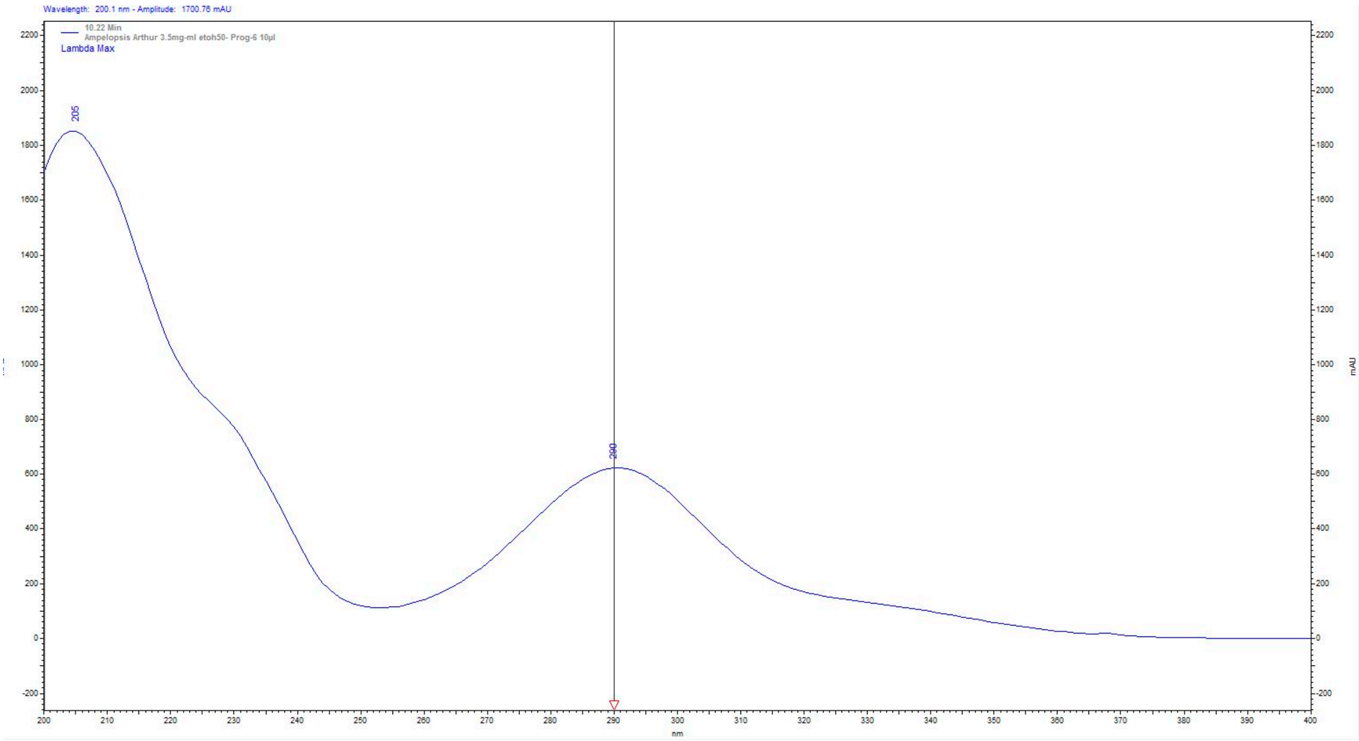

Figure S2 : DHM spectrum (200-400 nm)
